# EB1 protein alteration characterizes sporadic but not ulcerative colitis associated colorectal cancer

**DOI:** 10.18632/oncotarget.18978

**Published:** 2017-07-04

**Authors:** Timo Gemoll, Sophie L. Kollbeck, Karl F. Karstens, Gia G. Hò, Sonja Hartwig, Sarah Strohkamp, Katharina Schillo, Christoph Thorns, Martina Oberländer, Kathrin Kalies, Stefan Lehr, Jens K. Habermann

**Affiliations:** ^1^ Section for Translational Surgical Oncology and Biobanking, Department of Surgery, University of Lübeck and University Hospital Schleswig-Holstein, Campus Lübeck, D-23538 Lübeck, Germany; ^2^ Institute of Clinical Biochemistry and Pathobiochemistry, German Diabetes Center at the Heinrich-Heine-University Düsseldorf, Leibniz Center for Diabetes Research, D-40225 Düsseldorf, Germany; ^3^ German Center for Diabetes Research (DZD), D-85764 München-Neuherberg, Germany; ^4^ Department of Pathology, University Hospital Schleswig-Holstein, Campus Lübeck, D-23538 Lübeck, Germany; ^5^ Institute of Anatomy, University of Lübeck, D-23538 Lübeck, Germany

**Keywords:** ulcerative colitis associated colorectal cancer, EB1, two-dimensional gel electrophoresis, mass spectrometry, colorectal cancer

## Abstract

**Background:**

While carcinogenesis in Sporadic Colorectal Cancer (SCC) has been thoroughly studied, less is known about Ulcerative Colitis associated Colorectal Cancer (UCC). This study aimed to identify and validate differentially expressed proteins between clinical samples of SCC and UCC to elucidate new insights of UCC/SCC carcinogenesis and progression.

**Results:**

Multiplex-fluorescence two-dimensional gel electrophoresis (2-D DIGE) and mass spectrometry identified 67 proteoforms representing 43 distinct proteins. After analysis by Ingenuity Pathway Analysis^®^ (IPA), subsequent Western blot validation proofed the differential expression of Heat shock 27 kDA protein 1 (HSPB1) and Microtubule-associated protein R/EB family, member 1 (EB1) while the latter one showed also expression differences by immunohistochemistry.

**Materials and Methods:**

Fresh frozen tissue of UCC (*n* = 10) matched with SCC (*n* = 10) was investigated. Proteins of cancerous intestinal mucosal cells were obtained by Laser Capture Microdissection (LCM) and compared by 2-D DIGE. Significant spots were identified by mass spectrometry. After IPA, three proteins [EB1, HSPB1, and Annexin 5 (ANXA5)] were chosen for further validation by Western blotting and tissue microarray-based immunohistochemistry.

**Conclusions:**

This study identified significant differences in protein expression of colorectal carcinoma cells from UCC patients compared to patients with SCC. Particularly, EB1 was validated in an independent clinical cohort.

## INTRODUCTION

Patients with long-standing Ulcerative Colitis (UC) are at 2.4-fold increased risk for colorectal carcinoma via mechanisms that remain incompletely understood [1]. Although incidence of Ulcerative Colitis associated Cancer (UCC) has decreased with the help of preventive colonoscopy and advanced UC treatment [2], it is still the primary cause of death in those patients [3]. Clinically, UCCs show features divergent from SCC, e.g. younger age at onset, higher frequency of simultaneous tumors, and widespread, flat mucosa within large fields of genetic abnormalities [4–6]. Due to the challenging distinction from the surrounding inflammatory tissue, early endoscopic detection of UCC is difficult.

Riddel et al. postulated that UCCs do not develop through the adenoma-carcinoma-sequence but follow a colitis-dysplasia-carcinoma-sequence [7–9]. In this context, several investigations have identified genomic differences between SCC and UCC and proved divergence by Comparative genomic hybridization (CGH) analysis, loss of heterozygosity, number of amplifications, and altered genes [4, 10–13]. However, aneuploidy and genetic abnormalities, e.g. mutations in the adenomatous polyposis coli (*APC*), tumor protein 53 (*TP53*), B-cell lymphoma 2 (*BCL-2*) and V-Ki-ras2 Kirsten rat sarcoma viral oncogene homolog (*KRAS*) genes, exist in both, SCC and UCC, but with different frequency and timing [14–19]. On protein level, comparative analyses were derived from paraffin embedded tissues or cell culture and described different regulations in Heat shock protein 47, Toll-like receptor 4, β-Catenin, CD44, Claudin-1 and Claudin-2 [20–23] between SCC and UCC. However, no data have been published yet that evaluate intact proteins from fresh frozen SCC and UCC samples with high tumor representativity. Most clinical proteomics studies do not include representative tumors and thus profile samples with different ratios between tumor cells and stroma and apoptotic/necrotic cells. One possibility to procure highly representative sub-populations of cells from complex heterogeneous tissue samples is to use Laser Capture Microscopy (LCM). In combination with fluorescence-based multiplex two-dimensional gel electrophoresis (2-D DIGE), intact proteins inclusive their isoforms, alternative splice variants and post-translational modifications are detectable and quantifiable [24].

**Figure 1:**
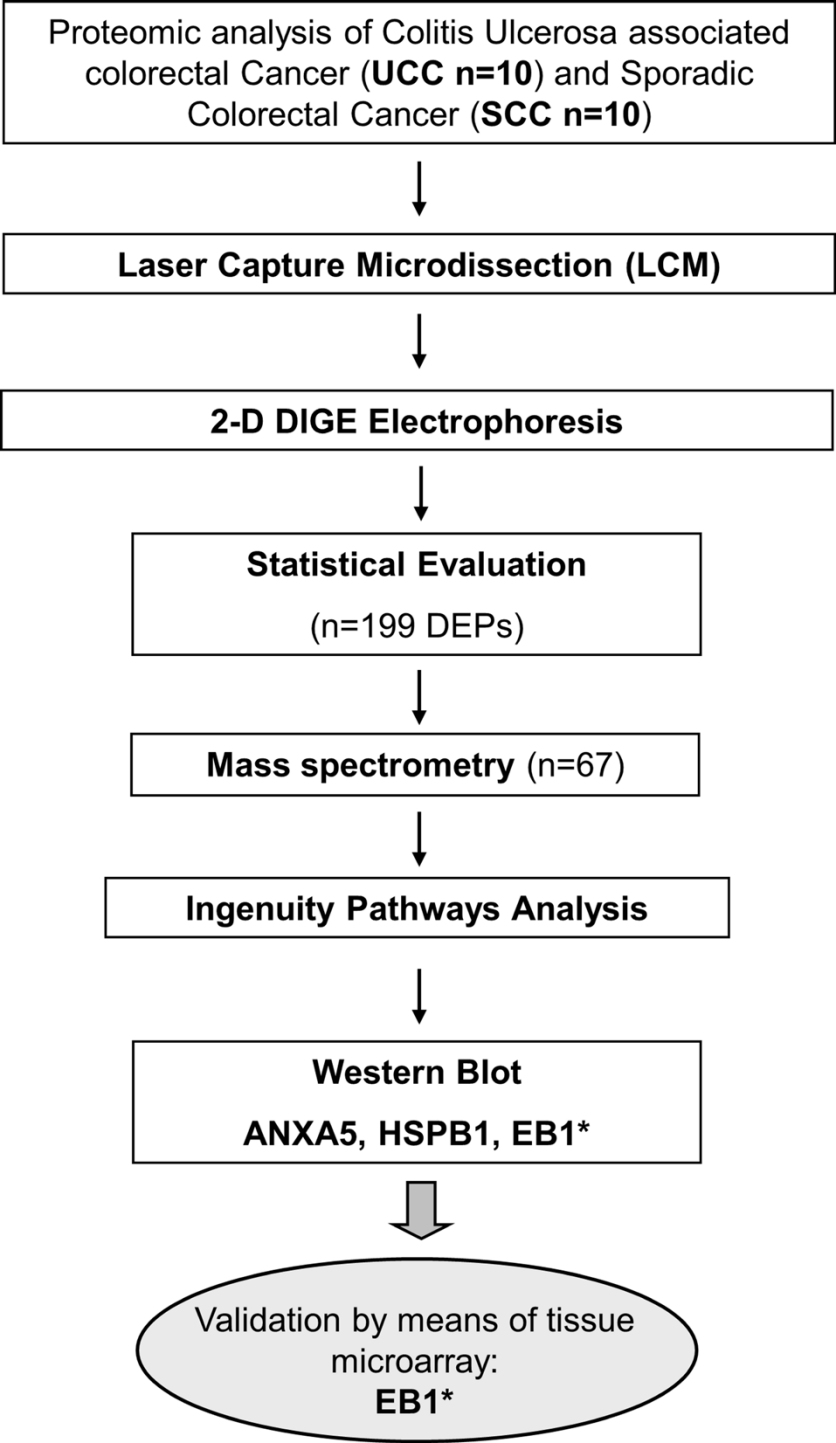
Overall workflow of the study design *target reached significance in individual validation steps. DEP = differential expressed protein.

Against this background, we performed LCM on fresh frozen samples in order to analyze the proteome of UCC and SCC specimens by means of 2-D DIGE, mass spectrometry and pathway analysis. Identified candidate proteins were further validated using Western blot and immunohistochemistry of clinical tissues compiled on a microarray. For overall study design, please see Figure 1.

## RESULTS

### 2-D DIGE analysis

**Figure 2:**
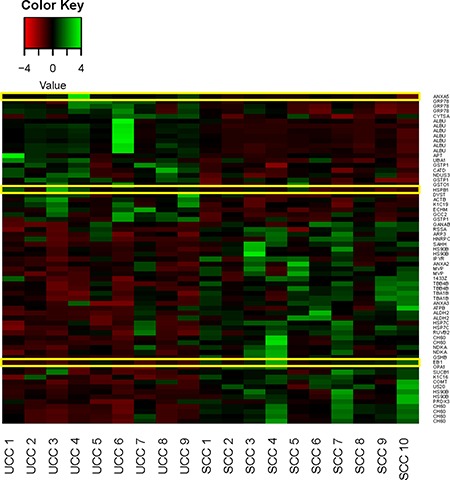
Heat map of normalized expression values for the identified proteins differentially expressed between Ulcerative Colitis associated Colorectal Cancer (UCC) and Sporadic Colorectal Cancer (SCC) samples Samples for each group are shown. Yellow outlines indicate targets for downstream validation studies. Green, high expression; red, low expression.

The number of the commonly detected spots in all gels was 1,030. Hereof, 199 spots showed statistical significance (*p* < 0.05) including a fold change between 1.18 and 2.95 (Supplementary Table 1). Hierarchical clustering of samples showed high discriminating potential and clear separation between the SCC and UCC group (Figure 2). Of the differentially expressed proteomforms, 67 were identified by MALDI mass spectrometry belonging to 43 distinct proteins. 26 proteins were upregulated and 41 were downregulated in UCC compared to SCC. Exemplary gel and spot images are depicted in Supplementary Figure 1. Mass spectrometry data of the identified spots are given in Supplementary Table 2.

### Functional interpretation and networking of proteins

**Figure 3:**
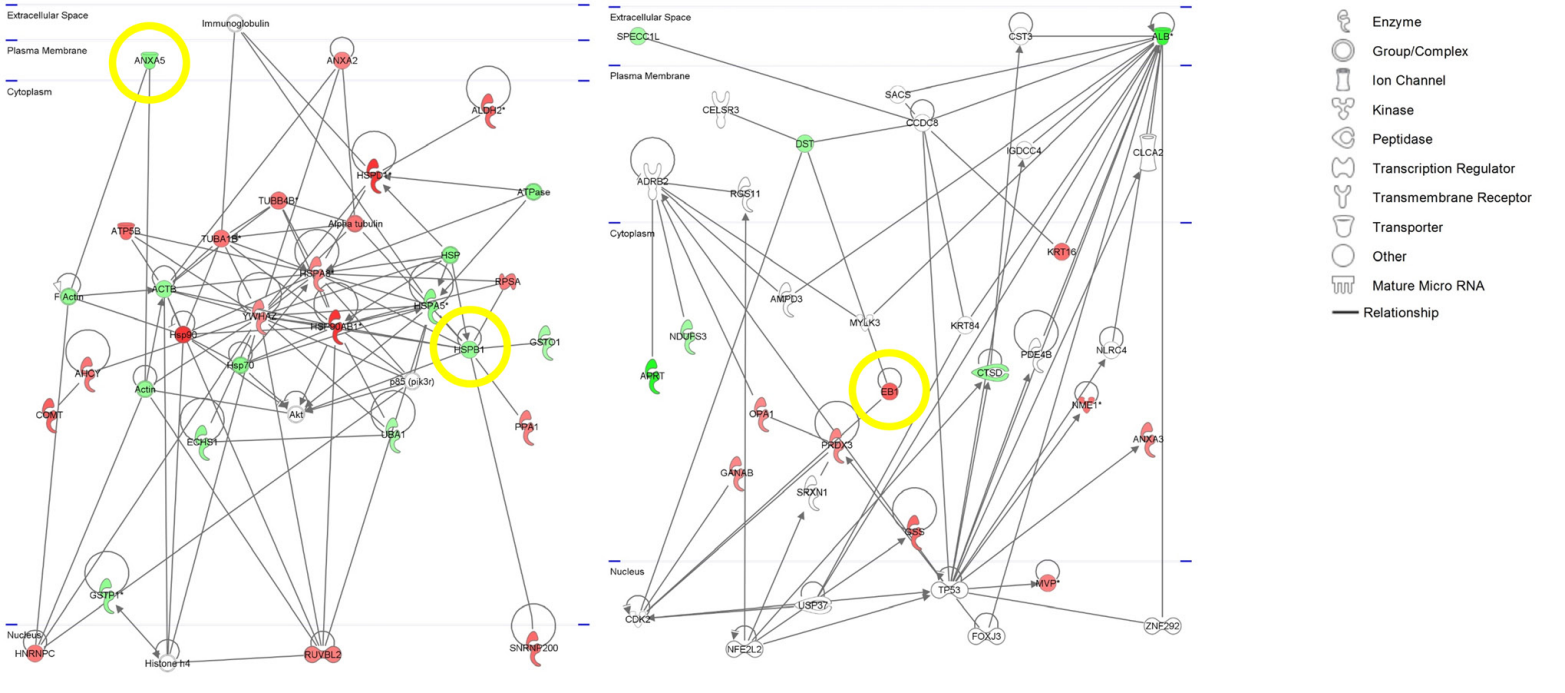
IPA-based pathway networks of differentially expressed proteins between ulcerative colitis associated colorectal cancer (UCC) and sporadic colorectal cancer (SCC) Red and green designations indicate up- and down-regulated proteins in the SCC compared to the UCC group. Yellow circles indicate targets for downstream validation studies.

The Ingenuity Pathways Knowledge Base was used to reveal different locations, functions and processes of identified proteins. Regarding the cellular location, the majority of the proteins were located in the cytoplasm (75%). The major biological process categories included cell growth and proliferation, cellular movement, post-translational modification, protein folding and DNA replication, recombination, and repair. IPA analysis of functional associations pointed to three significant networks: Network 1–Neurological Disease, Psychological Disorders, Post-Translational Modification (score 60), Network 2–Gastrointestinal Disease, Hepatic System Disease, Metabolic Disease (score 32), and Network 3–Cell Signaling, Cellular Assembly and Organization, Dermatological Diseases and Conditions (Score 8) (Figure 3). Network 1 with 24 proteins (ACTB, AHCY, ALDH2, ANXA2, ANXA5, ATP5B, COMT, ECHS1, GSTO1, GSTP1, HNRNPC, HSP90AB1, HSPA5, HSPA8, HSPB1, HSPD1, PPA1, RPSA, RUVBL2, SNRNP200, TUBA1B, TUBB4B, UBA1, YWHAZ), network 2 with 15 proteins (ALB, ANXA3, APRT, CTSD, DST, GANAB, GSS, KRT16, EB1, MVP, NDUFS3, NME1, OPA1, PRDX3, SPECC1L), and network 3 with five proteins (ACTR3, GCC2, KRT19, NME1, SUCLA2) are associated with cancer as top disease (*p* < 0.022) as well as with v-myc avian myelocytomatosis viral oncogene homolog (MYC, *p* < 0.001) as upstream regulator. Our set of proteins was further analyzed using the IPA biomarker filter which allows matching the input protein list with known disease profiles and lists of biomarkers known for a disease. Selecting large intestine cancer and colon cancer cell lines as filtering criteria, 34 of the proteins (ACTB, ACTR3, AHCY, ALDH2, ANXA2, ANXA3, ANXA5, ATP5B, CTSD, GANAB, GCC2, GSS, GSTO1, GSTP1, HNRNPC, HSP90AB1, HSPA5, HSPA8, HSPB1, HSPD1, KRT19, EB1, MVP, NDUFS3, NME1, OPA1, PPA1, PRDX3, SPECC1L, SUCLA2, TUBA1B, TUBB4B, UBA1, YWHAZ) were identified as markers associated with colon cancer. Top canonical pathways, diseases and functions are summarized in Supplementary Table 3. All targets were evaluated for their biological function by an individual IPA database search. Cancer- and inflammation-relevant candidates were subsequently subjected to a Pubmed-based literature search using the following term: (”x”[tiab]) AND (cancer[tiab] OR tumor[tiab] OR carcinoma[tiab]) AND Humans[Mesh] AND English[lang]), whereby “x” stands for each candidate after IPA search. Bibliographies of the articles discovered were additionally checked for relevant citations.

**Figure 4:**
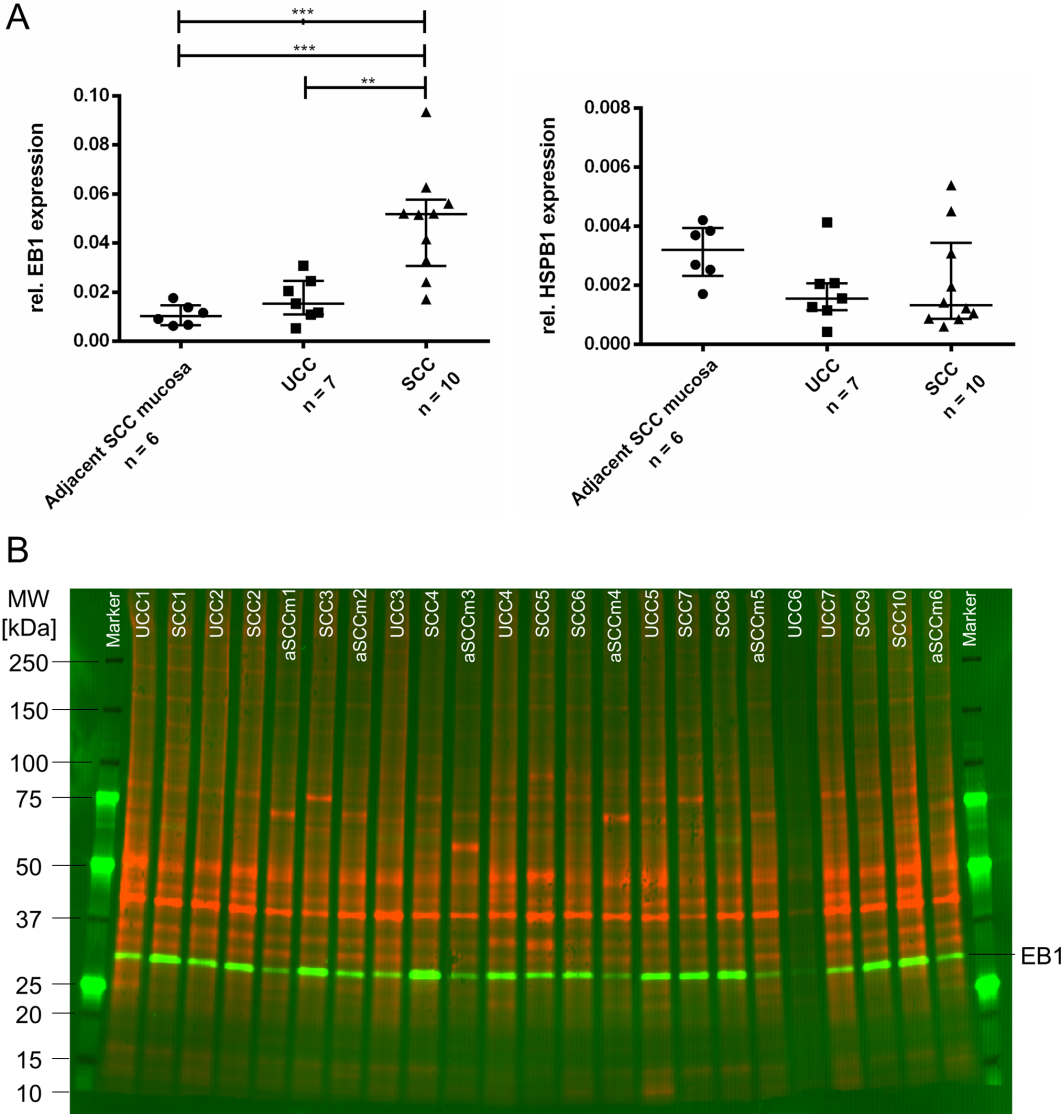
(**A**) Western blot results of validated target proteins EB1 (left) and HSPB1 (right). EB1 is significantly lower expressed in healthy controls whereas HSPB1 showed a trend to be higher expressed in healthy controls (***0.0001 < *P* < .001; **0.001 < *P* < 0.01). Plots show relative protein expressions (volume with normalization against total protein) of each sample as well as median and interquartile range of each group. (**B**) Representative multiplex Western blot image of EB1. While the total proteome used for normalization is presented in red (G-Dye 300), the detected protein EB1 is displayed in green colors (G-Dye 200). SCC, Sporadic Colorectal Cancer; UCC, Ulcerative Colitis associated colorectal Cancer; aSCCm, adjacent Sporadic Colorectal Cancer mucosa; MW, molecular weight.

### Validation of selected proteins by Western blot and immunohistochemistry

Based on IPA analysis, expression differences, biological function and literature search, Microtubule-associated protein R/EB family, member 1 (EB1), Heat shock 27 kDA protein 1 (HSPB1), and Annexin 5 (ANXA5) were further evaluated by Western blot using the same patient cohort as for 2-D DIGE plus additional six normal mucosa samples of SCC patients. The measured band intensities of tested proteins were exactly normalized to the total protein and displayed as relative intensities. The results confirmed the abundance levels for EB1 and HSPB1 obtained by the 2-D DIGE experiment (Figure 4): The protein levels of EB1 in the SCC group were significantly higher than in the UCC group (*p* = 0.0019). EB1 expression in samples of healthy controls were slightly lower than in UCC (not significant) and significantly lower compared to SCC (*p* = 0.0005). The levels of HSPB1 were lower in SCC compared to UCC and showed higher values in healthy controls compared to UCC and SCC. Both comparisons did not reach significance. ANXA5 was not congruent in its expression with the initial 2-D DIGE data (data not shown).

**Figure 5:**
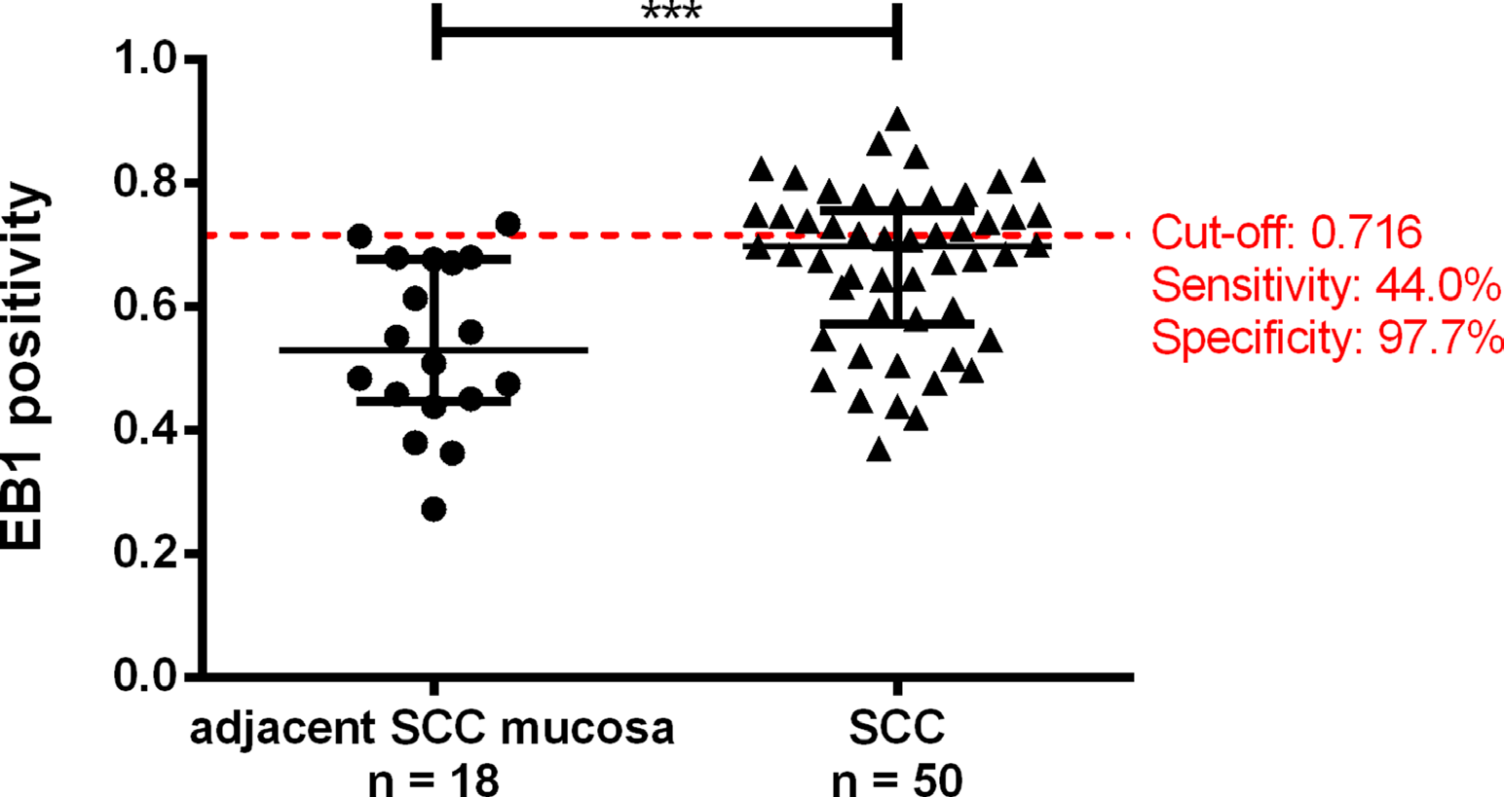
Tissue-microarray-based immunohistochemical evaluation of EB1 by means of Image scope comparing adjacent Sporadic Colorectal Carcinoma mucosa (aSCCm) and Sporadic Colorectal Carcinomas (SCC) Red line represents the cut-off value for the normal mucosa and SCC comparison with highest sensitivity and specificity (left). (***0.0001 < *P* < 0.001).

In order to further validate the EB1 expression changes in SCC compared to normal mucosa in an independent larger clinical cohort, TMA sections were evaluated by IHC. Immunopositivity (IP) of 68 stained samples revealed EB1 with a significant stronger positivity in SCC (median: 0.6990) compared to normal mucosa (median: 0.5294; *p* = 0.0007; Figure 5). Exemplary stainings are presented in Supplementary Figure 2. Within the SCC group, no association between EB1 immunopositivity and clinicopathological parameters (positive versus negative for metastasis, UICC I/II versus UICC III/IV cancers, and patients with a survival of less versus more than 60 months; Supplementary Figure 3) were observed. Best combination of sensitivity and specificity for EB1 differentiating SCC from normal mucosa was 44.0 % and 97.7 %, respectively (cut-off: 0.716; Figure 5). Manual scoring of the staining confirmed these findings.

## DISCUSSION

This study identified differential expressed proteins between SCC and UCC while elucidating insights of SCC and UCC carcinogenesis and progression.

In this context, significant differences in protein expression between samples with SCC and UCC were detected by multiplex-fluorescence two-dimensional gel electrophoresis (2-D DIGE). To overcome the problem of tissue heterogeneity and to improve accuracy by measuring proteomic changes directly within malignant and healthy cells, upfront sample enrichment using Laser Capture Microdissection (LCM) was performed. Mass spectrometric identification revealed in total 67 proteoforms representing 43 unique proteins that interact in signal pathways of cell growth and maintenance, energy pathways, and metabolism. Five of the identified proteins showed at least three discriminative isoforms and underlined the potential of the 2-D DIGE approach in detecting different activity status of molecules. HSP90, for example, was identified four-times and is known to quickly adapt to changes in the intracellular environment through post-translational modifications [25].

Two proteins (EB1 and HSPB1) proofed their differential expression also by Western blot and tissue microarray-based immunohistochemical analyses: while EB1 was validated significantly, HSPB1 did not reach significance but showed the same trend as observed in 2-D DIGE experiment. While the latter might potentially be due to a limited antibody specificity, Western blot of ANXA5 showed no significance between groups and did not correlate with 2-D DIGE expression data.

HSPB1 (located on chromosome 7q11.23) is a member of the heat shock protein (HSP) family that have been defined as proteins induced by heat shock and other environmental stress and developmental changes. Upon stress induction, HSPB1 translocates from the cytoplasm to the nucleus and is implicated in protein-protein interactions, e.g. folding, translocation, and aggregation. During a stressed cell state, HSPB1 functions as a chaperone, while in an unstressed state, it is thought to provide cytoskeletal structural stability [26]. Many of the functions of HSPB1 suggest important roles in several cancers including stomach, breast, ovary and prostate [27]. Overexpression of HSPB1 was found to be associated with poor prognosis for patients with meningioma [28]. Moreover, it has been shown that HSPB1 accumulation reduces the apoptotic process induced by alkylating agents in colorectal cancer cells [29]. So far, HSPB1 has not been evaluated in studies compromising UCCs. By means of 2-D DIGE, we could now show a higher expression of HSPB1 in UCCs compared to SCCs which was confirmed in trend by Western blotting. Interestingly, HSPB1 upregulation is associated with the appearance of mutations in the *TP53* gene [30]. As this mutation is an early event during UCC carcinogenesis [31], our results are in line with the literature and stress the hypothesis of different pathways including different timing of gene mutations during UCC and SCC carcinogenesis. Our study showed highest expression levels in normal colonic tissue which stands in contrast to previous studies [27]. However, published data did not evaluate proteins from fresh frozen SCC and UCC samples with high tumor representativity using Laser Capture Microscopy (LCM).

In contrast to the higher expression of HSPB1 in UCCs, EB1 was higher expressed in SCCs. EB1 is a binding protein of the tumor suppressor gene *APC* (adenomatous polyposis coli) which is known to play an important role during colorectal cancer development [32]. The elevated expression of EB1 was previously reported in several malignancies including liver cancer [33, 34], gastric carcinoma [35], esophageal squamous cell carcinoma [36], and breast cancer [37]. Besides these, the expression differences of EB1 between SCC and UCC has not been investigated prior to the present report. For SCC, Sugihara and colleagues suggested that EB1 was overexpressed in tumor cells in correlation with poor prognosis [38]. In our study applying now LCM coupled with 2-D DIGE, the protein level of EB1 was significantly lower in UCCs compared to SCCs. Subsequent quantitative Western Blot analysis confirmed these findings (*p* = 0.0019) and detected additionally a strong enhancement in SCC compared to normal intestinal mucosa (*p* = 0.0005). EB1 levels in UCCs were higher by trend compared to normal mucosa. Our TMA-based validation including software-based evaluation supported high levels of EB1 in SCC: while EB1 immunoreactivity was weak or absent in normal mucosa, carcinomas showed moderate to strong immunoreactivity (*p* = 0.0007; Figure 5).

It was previously proposed that the APC-EB1 interaction regulates the mitotic spindle, chromosome alignment and cellular proliferation [39, 40]. Hence, dysregulation of the APC-EB1, e.g. through APC mutation and/or EB1 overexpression, may promote spindle defects and aberrant chromosomal segregation which in turn initiates cancer development and progression. With *APC* mutations as an early event in SCC carcinogenesis, it seems therefore reasonable to suggest that APC-EB1 relations are abrogated which results in a higher protein level of unbound EB1. The abrogation of APC-EB1 interactions including the contribution to cancer progression has been also speculated earlier [32, 41, 42]. Although the mutational APC status of the sample cohort was not available, newly acquired *APC* mutations are rare for UCCs [43] and thus may increase the probability of undisturbed APC-EB1 interactions and a lower amount of unbound EB1. In combination with a minor higher expression in UCCs compared to normal mucosa, our data supported the evidence that the carcinogenesis pathway in UCCs is distinct to that of SCCs. From a histological perspective, sporadic tumors tend to follow the adenoma-carcinoma-sequence with the stepwise accumulation of genetic mutations in onco and tumor suppressor genes [44]. However, colitis-associated colorectal cancer progress through the pathway of low- (LGD) and high-grade dysplasia (HGD) to carcinoma and is less well explored with significant differences in the requirement and timing of genetic and epigenetic alterations [45]. Profound differences of UCC and SCC carcinogenesis were supported just recently by Yaeger et al., who found different genomic variances in UCCs compared to SCCs by next-generation-sequencing [43]. In addition, translational regulation of genes might contribute to the different cancer development pathways: although Habermann et al. described a chromosomal gain of 20q11 to 20q13 in both SCC and UCC by means of CGH [13], a higher EB1 (located on chromosome 20q11.21) expression on protein level was not detected in UCCs in the current study. Interestingly and although chemotherapy response in UCCs results less often in complete response or stable disease (Engelhardt et al., 2011), standard therapy regimes do not differ from those for SCCs (e.g. fluorouracil, irinotecan, oxaliplatin). Advanced individual therapy concepts focus on well characterized mutations, e.g. *KRAS* and *BRAF*, and thus stress the understanding of tumor biologies for individualized medicine. In this context, our results might present valuable information to individualize UCC from SCC therapies in the future.

In summary, EB1 and HSPB1 were detected as differential expressed proteins between UCC and SCC. To our knowledge, this is the first study that systematically identified and validated differentially expressed proteins between UCC and SCC with respect to enriched tumor representativity. The results provide novel insights of the carcinogenesis and progression of SCC and UCC.

## MATERIALS AND METHODS

### Patients and tissue samples

The analyzed material consisted of 20 surgically resected colorectal tumors obtained from ten patients surgically treated for SCC and ten patients operated for UCC at the Department of Surgery, University Hospital Schleswig-Holstein, Campus Lübeck. In addition, six samples of adjacent normal intestinal mucosa of SCC patients were obtained for evaluation by Western blotting. An in-house compiled tissue microarray (TMA) contained independent samples of sporadic carcinomas at different tumor stages (*n* = 60) and adjacent mucosa (*n* = 30). All clinicopathological data of both patient series are presented in Table 1A and 1B. The study was approved by the local Ethics Committee of the University of Lübeck (#07–124).

**Table 1A:** Patient cohort of the two-dimensional gel electrophoresis and western blot

Clinical parameter	Healthy controls^a^	UCC^b^	SCC
Sex [male/female]	2/4	4/5	6/4
Age	71.8	43.7	68.9
UICC I		1	2
UICC II		3	3
UICC III		5	5
Grading [1/2/3]		0/3/6	0/9/1

^a^Healthy controls were obtained from adjacent non-neoplastic mucosa and were not included in the 2-D DIGE analysis

^b^Two UCC samples (male, 36 years, UICC II, grading 3 and male, 37 years, UICC II, grading 2) could not be included in the Western blot analysis

SCC: Sporadic Colorectal Cancer; UCC: Ulcerative Colitis associated Colorectal Cancer

**Table 1B:** Patient cohort of the in-house compiled tissue microarray of Sporadic Colorectal Cancer (SCC) and adjacent normal colon tissues set as healthy controls

Clinical parameter	Healthy controls	SCC
Sex [male/female]	16/14	30/30
Age	56.7	68.7
UICC I		7
UICC II		23
UICC III		30
Grading [1/2/3]		3/41/16
5-year status [alive/death]		33/27
Survival [months]		0.5–183.1

### Laser capture microdissection (LCM)

Cancerous and normal intestinal mucosal cells were obtained by Laser Capture Microdissection (LCM). LCM technique was optimized according to previous reports [46, 47]. 40 μm-thick frozen sections were cut from tumor tissues and stained by toluidine blue. Cancerous and non-neoplastic adjacent SCC mucosa cells were recovered under microscopic observation with the assistance of an ultraviolet laser (Zeiss Axiovert 200M, Palm Microbeam; Focus 63–64; Energy 79–84; LPC 100). A 3.2 × 10^9^ mm^2^ tissue area was collected for each sample. The recovered cells were lysed in RLT buffer (Qiagen, Germantown, USA) and proteins were extracted by means of the AllPrepMini kit (Qiagen). Samples were purified with the ReadyPrep 2-D Cleanup Kit (Bio-Rad Laboratories, Hercules, USA), diluted in 20 μl DIGE buffer [30 mmol/L TRIS, 7 mol/L urea, 2 mol/L thiourea, 4% (w/v) CHAPS] and stored at −80°C until further use. Protein concentration was determined by the fluorescence-based EZQ-Quantitation Kit (Life Technologies, Carlsbad, USA).

### Multiplex fluorescent two-dimensional gel-electrophoresis (2-D DIGE)

Proteins for 2-D DIGE analysis were labeled with the Refraction-D^™^ labeling kit (NH DyeAGNOSTIC, Halle, Germany). While a total of 50 μg protein per sample was mixed with Tris-HCl (1.5 mol/L, pH 8.8) and 50 nmol/L G-100 or G-200, respectively, a pooled internal standard (50 μg) for exact quantification was set up of Tris-HCl (1.5 mol/L, pH 8.8) and 50 nmol/L G-300. After incubation in darkness at 4°C for 30 min, each reaction was terminated by adding 10 mmol/L lysine on ice for 10 min. Samples and internal standard were combined and diluted with rehydration sample buffer [7 mol/L urea, 2 mol/L thiourea, 2% (w/v) CHAPS, 2% (v/v) ampholytes (pH 4–7, SERVA Electrophoresis, Heidelberg, Germany) and a trace of bromophenol blue] to a final volume of 450 μl. 24 cm Immobiline Dry Strips^™^ (pH 4–7, GE Healthcare, Illinois, USA) were used for isoelectric focusing which was carried out in a Protean^®^ i12™ IEF cell (Bio-Rad Laboratories) at 20°C reaching approximately 57,700 Vhs. One UCC sample with one matched SCC sample and the pooled standard were applied per gel strip. After equilibration (Buffer Kit for 2D HPE™ Gels, SERVA Electrophoresis), horizontal second dimension was executed using precast plastic-backed 12.5% acrylamide gels (2DHPE™ Large Gel NF 12.5% Kit, 0.65 × 200 × 255 mm, SERVA Electrophoresis). 2-D DIGE images were acquired using a Typhoon FLA 9000 scanner (GE Healthcare). Detected spots were matched and analyzed using Progenesis SameSpots^®^ (v4.1, Nonlinear Dynamics, Newcastle, UK). Briefly, all 2-D DIGE images are grouped according to the gels and aligned to a selected reference image. Alignment at the pixel level removed any positional variation introduced during the electrophoresis and imaging processes and thus provides a direct and accurate comparison. Spots were automatically co-detected on all images in the analysis and normalized in their spot volume against the applied internal standard. Spots that showed significant expression differences (*p* < 0.05) between groups were picked using a robotic spot picker (GE Healthcare). Based on statistical outlier calculation, one UCC sample was detected and subsequently excluded from further analysis.

### Mass spectrometric protein identification

Gel plugs were washed alternating in 25 mmol/L ammonium bicarbonate and 25 mmol/L NH_4_HCO_3_ in 50% (v/v) acetonitrile. Neat Acetonitril was added and removed. Dried gel plugs were rehydrated in 7 μl icecold solution of 3.5 ng/μl sequencing grade trypsin (Promega, Madison, USA) in 25 mmol/L NH_4_HCO_3_ 2%ACN. Proteins were digested in-gel at 37°C overnight (16 h). Peptides were extracted for 60 min with 7 μl of 1% trifluoroacetic acid (TFA) and directly applied to a Prespotted AnchorChip MALDI target (Bruker Daltonics, Bremen, Germany) according to the manufacturer's instructions. Subsequently, samples were analyzed in an Ultraflex MALDI-TOF/TOF mass spectrometer (Bruker Daltonics). Peptide mass fingerprint spectra were accumulated by 600 shots with a resolution higher than 2,000 based on Centroid peak detection algorithm. Acquired mass spectra were automatically calibrated and annotated using FlexAnalysis as part of the Compass 1.3 software (v2012, Bruker Daltonics). SNAP peak picking algorithm was used in the mass range of 850 to 3,900 m/z with a signal to noise ratio of 2.5. Calibration was carried out by using an internal mass calibration list of 91 labspecific contaminants masses. For protein identification, results from each individual protein spot were used to search a human subset in the Swiss-Prot_2012 (20,245 sequences) nonredundant database by means of Mascot search engine (v2.2, Matrix Science Ltd., London, UK) in consideration of the following settings: (i) enzyme “trypsin”, (ii) species “human”, (iii) fixed modifications “carbamidomethyl”, (iv) optional modifications “methionine oxidation” and (v) missed cleavages “1”. Mass tolerance was set to 100 ppm. Using these settings, a Mascot score > 52 was taken as significant (*p* ≤ 0.05).

### Functional analysis of identified proteins

Ingenuity pathway analysis (IPA, Qiagen) was used to determine the connectivity between identified proteins, their biofunction and localization. Only direct relationships were included in the analysis. Each protein symbol was mapped to its own protein object in the Ingenuity Pathways Knowledge Database. Protein identities showing different expression trends between SCC and UCC among their proteoforms were excluded. Based on unsupervised IPA and on literature review we selected three proteins (EB1, ANXA5, HSPB1) for further validation using quantitative Western blot and immunohistochemical analysis.

### Quantitative fluorescence-based multiplex western blotting (qWB)

Quantitative western blotting was performed by using total protein normalization. Total protein was pre-labeled with G-Dye-300 fluorescence dyes (NH DyeAgnostic) according to manufacturer's recommendations and separated by sodium dodecyl sulfate (SDS)-Page (Criterion™ TGX™ precast Gel, 4–12%; Bio-Rad Laboratories) at constant 200 V for 35 min in a Criterion™ Vertical Electrophoresis Cell (Bio-Rad Laboratories). Labeled proteins were transferred onto a PVDF membrane (Immobilon^®^-FL PVDF, 0.45 μm, Merck Millipore, Billerica, USA) using a Trans-Blot^®^ Turbo™ Transfer System (Bio-Rad Laboratories). Membranes were blocked at room temperature for 1 h with 2 % Amersham ECL Prime Blocking Agent (GE Healthcare), dissolved in 1 × TBS with 0.1 % Tween-20 (pH 7.6, Cell Signaling, Danvers, USA) and incubated with monoclonal primary antibodies against anti-ANXA5 (#TA307564, anti-rabbit, 1:1,000; OriGene Technologies, Rockville, USA), anti-EB1 (#SC-47704, anti-mouse, 1:100; Santa Cruz Biotechnology, Dallas, USA) and anti-HSPB1 (Ab00314–1.1, anti-mouse, 1:1,000; Absolute Antibody, Oxford, UK). Blots were incubated for 1 h at room temperature with Cy3-labled goat-anti-mouse or goat-anti-rabbit secondary antibodies (Amersham ECL^TM^ Plex CyDye-Conjugated Antibodies, GE Healthcare) diluted 1:2,500 in 2% blocking buffer. Final protein fluorescence visualization at 532 nm and 648 nm was carried out with a Typhoon FLA 9000 laser scanner (GE Healthcare). Densitometric analyses of loaded total protein and antibody-targeted protein bands were performed using the ImageQuant TL software (GE Healthcare). Each specific antibody-targeted protein band (532 nm channel detection) was normalized against the loaded total protein (648 nm channel detection). The density of a given protein band was measured as the total volume under the three-dimensional peak. Background subtraction was set to rolling ball for antibody-targeted protein bands. After 2-D DIGE profiling, two additional UCC samples showed an insufficient protein amount for Western Blot validation and were thus excluded from downstream experiments.

### Tissue microarray of healthy controls and SCCs

Immunohistochemical analysis was performed on 4 μm sections of a formalin-fixed, paraffin-embedded tissue microarray (TMA) of an independent cohort of 60 colorectal carcinomas as well as 30 corresponding adjacent normal mucosa specimens as described previously [48]. All colorectal cancer samples were equally subdivided into carcinomas with lymph node positive and negative metastasis, UICC I/II and UICC III/IV cancers, and patients with a survival of less and more than 60 months (Table 1B). Sections were incubated with a primary antibody against EB1 (1:10,000, monoclonal, Santa Cruz Biotechnology) overnight at 4°C. Staining was performed using the avidin-biotin complex (ABC) and counterstaining with hematoxylin, followed by dehydration and mounting. Immunopositivity of EB1 was analyzed using an automated computer system with positive pixel count: Digital microscopy (Pannoramic DESK, 3D Histech, Budapest, Hungary) scanned each slide from which histological representative regions were assessed quantitatively by Image Scope (v9.1, Aperio, Vista, USA). The methodical approach allowed the analysis of distinct cell populations of non-neoplastic epithelial crypts or tumor cells excluding stromal cells. One senior pathologist (C.T.) reviewed all slides after H&E staining. Immunopositivity of the molecular markers were collected as continues variables ranging from 0 to 1.

### Statistical analysis

2-D DIGE results with differences in expression levels between the two groups were analyzed with SameSpot^®^ software followed by hierarchical clustering. Statistical analysis of Western blotting and immunohistochemistry were performed by GraphPad Prism^®^. Mann-Whitney-U and Kruskal-Wallis tests were calculated with alternative hypotheses based on observed expression differences in 2-D DIGE gel data. For immunohistochemistry, duplicated TMA-cores per case were averaged. Kaplan-Meier curves were calculated and tested for significant differences by the logrank test. Fisher's exact and Mann-Whitney-U tests were used to compare sex and age, respectively. For each test, a significance level of 5% was used.

## SUPPLEMENTARY MATERIALS FIGURES AND TABLES







## Abbreviations

2-D DIGE: multiplex-fluorescence two-dimensional gel electrophoresis
ANXA5: Annexin 5
APC: adenomatous polyposis coli
BCL-2: B-cell lymphoma 2
CGH: Comparative genomic hybridization
EB1: microtubule-associated protein R/EB family, member 1
HSPB1: heat shock 27 kDA protein 1
IHC: immunohistochemistry
IPA: Ingenuity pathway evaluation
KRAS: V-Ki-ras2 Kirsten rat sarcoma viral oncogene homolog genes
LCM: Laser Capture Microdissection
PCA: principal component analysis
qWB: quantitative fluorescence-based multiplex western blotting
SCC: sporadic colorectal cancer
SDS: sodium dodecyl sulfate (SDS)-Page
TMA: tissue microarray
TP53: tumor protein 53 (TP53)
UCC: ulcerative colitis associated colorectal cancer
UICC: Union internationale contre le cancer
