# Supplementary material for: EB1 protein alteration characterizes sporadic but not ulcerative colitis associated colorectal cancer
Source: Oncotarget. 2017 Jul 4;8(33):54939–50. doi: 10.18632/oncotarget.18978 (PMC5589632; doi:10.18632/oncotarget.18978)
Supplement: Supplementary file 1 [file oncotarget-08-54939-s001.pdf]

# EB1 protein alteration characterizes sporadic but not ulcerative colitis associated colorectal cancer

## SUPPLEMENTARY MATERIALS

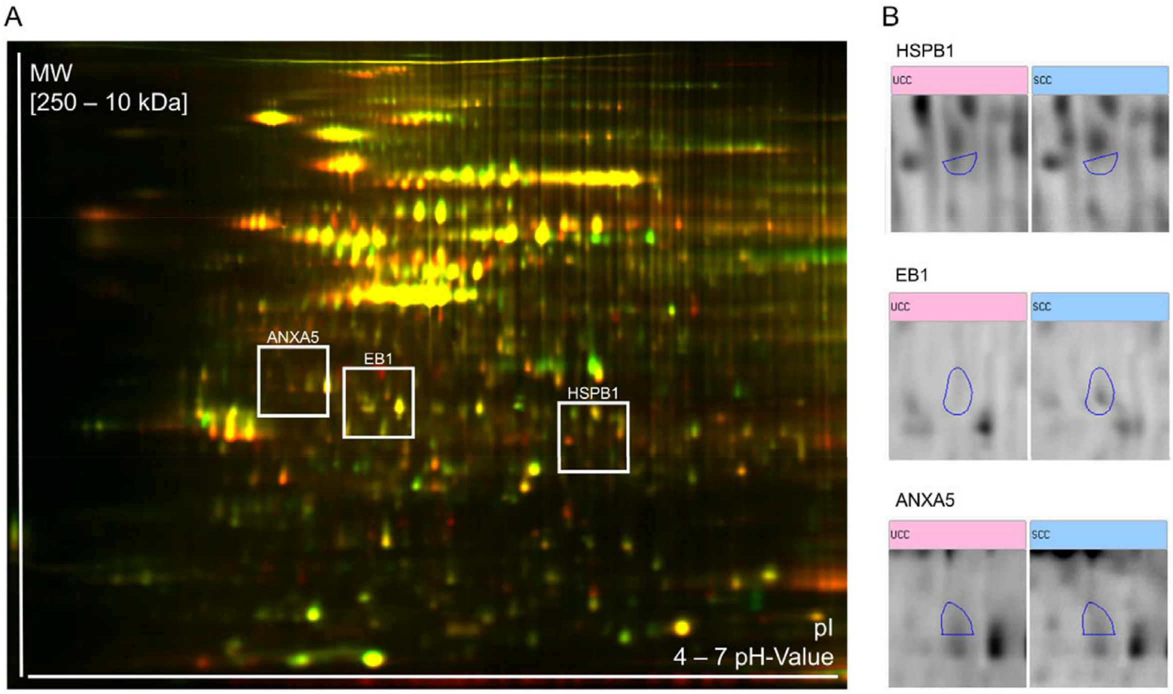


**Supplementary Figure 1:** (**A**) Exemplary multiplex gel image of one SCC (green) and one UCC sample (red) highlighting the identified proteins HSPB1, EB1, and ANXA5. (**B**) Magnified presentation of HSPB1, EB1, and ANXA5 showing one SCC and one UCC sample. UCC, Ulcerative Colitis associated Colorectal Cancer; SCC, Sporadic Colorectal Cancer; MW, molecular weight; pI, isoelectric point.

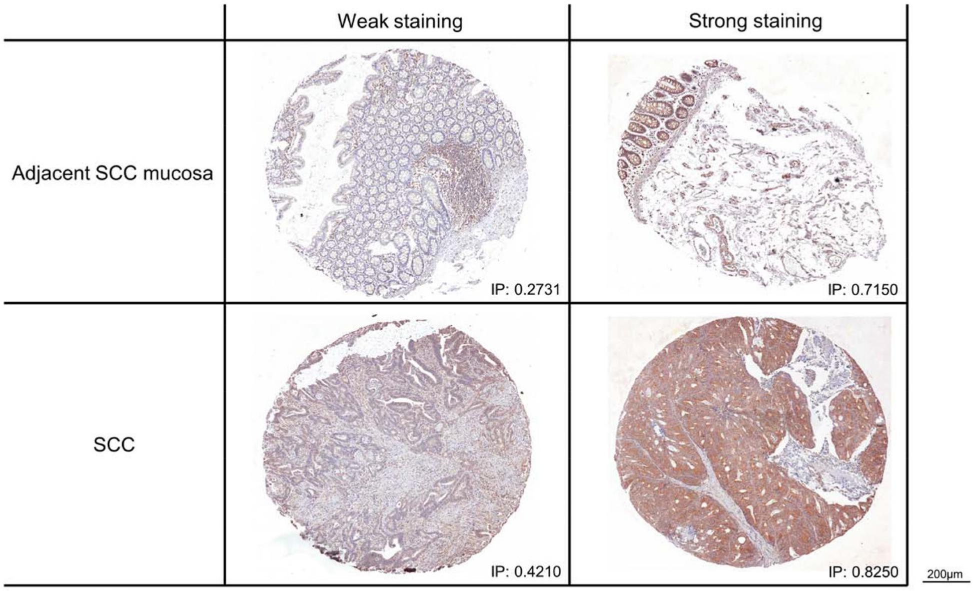


**Supplementary Figure 2: Exemplary immunohistochemical EB1 TMA-based stainings of 60 colorectal carcinomas as well as 30 corresponding adjacent normal mucosa specimens.** IP, immunopositivity.

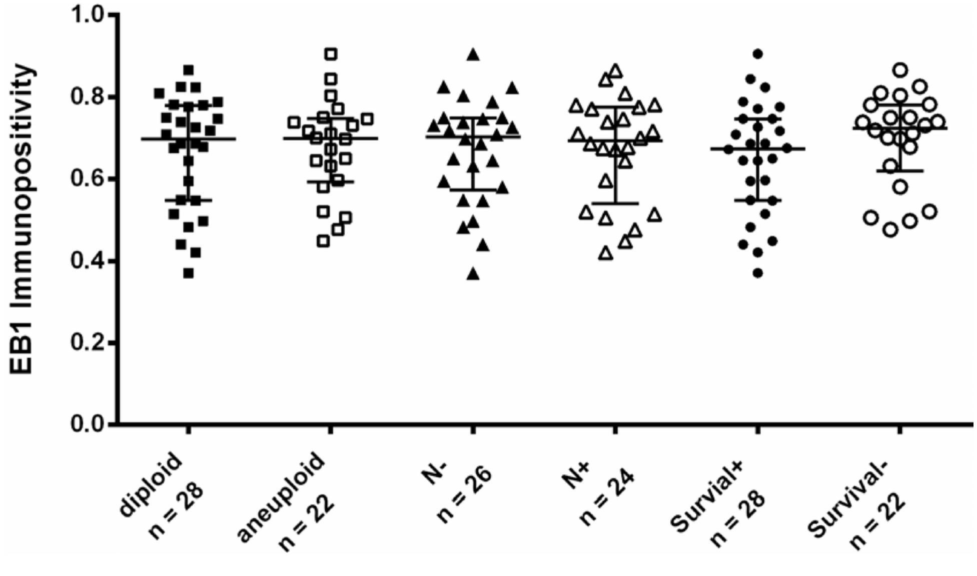


**Supplementary Figure 3: Tissue-microarray-based immunohistochemical evaluation of EB1 with respect to clinical parameters of SCCs.** N-/N+, lymph node positive and negative metastasis; Survival-/+, patients with a survival of less and more than 60 months.

**Supplementary Table 1: Raw data of the 2-D DIGE software evaluation.** See_Supplementary_Table 1

**Supplementary Table 2: Mass spectrometry data of 67 identified spots of the Ulcerative Colitis associated colorectal Cancer (UCC) vs. Sporadic Colorectal Cancer (SCC) comparison.** See_ Supplementary_Table 2

**Supplementary Table 3: Results of ingenuity® pathway analysis**

| Top networks | Score | Top diseases and disorders | *p*-Value | # of Molecules | Molecular and Cellular Functions | *p*-Value | # of Molecules |
|---|---|---|---|---|---|---|---|
| Neurological Disease, Psychological Disorders, Post-Translational Modification | 60 | Cancer | 1.22E-02–3.72E-14 | 34 | Cellular Growth and Proliferation | 1.03E-02–4.83E-09 | 29 |
| Gastrointestinal Disease, Hepatic System Disease, Metabolic Disease | 32 | Organismal Injury and Abnormalities | 1.23E-02 – 3.72E-14 | 38 | Cellular Movement | 1.03E-02–1.06E-08 | 21 |
| Cell Signaling, Cellular Assembly and Organization, Dermatological Diseases and Conditions | 8 | Neurological Disease | 1.16E-02–2.13E-12 | 24 | Post-Translational Modification | 8.23E-03–2.94E-08 | 11 |
| | | Psychological Disorders | 1.03E-02–2.13E-12 | 21 | Protein Folding | 7.72E-04–2.94E-08 | 6 |
| | | Gastrointestinal Disease | 1.03E-02–2.08E-11 | 27 | DNA Replication, Recombination, and Repair | 1.03E-02–4.72E-08 | 13 |
