# Supplementary material for: EB1 protein alteration characterizes sporadic but not ulcerative colitis associated colorectal cancer
Source: Oncotarget. 2017 Jul 4;8(33):54939–50. doi: 10.18632/oncotarget.18978 (PMC5589632; doi:10.18632/oncotarget.18978)
Supplement: Supplementary file 3 [file oncotarget-08-54939-s003.docx]

Supplementary Table 2: Mass spectrometry data of 67 identified spots of the Ulcerative Colitis associated colorectal Cancer (UCC) vs. Sporadic Colorectal Cancer (SCC) comparison

| **#** | **Uniprot ID** | **Protein name** | **Chromosomal location*** | **MW**  **[theor.]** | **pI**  **[theor.]** | **MS MS coverage (%)** | **Matched /**  **unmatched peaks** | **Mascot Score** | **P value** | **Fold-change (SCC/UCC)**** |
| --- | --- | --- | --- | --- | --- | --- | --- | --- | --- | --- |
| 1206 | 1433Z_HUMAN | Tyrosin 3-monooxygenase/tryptophan 5-monooxygenase | 8q23.1 | 28 | 4.60 | 55 | 19/111 | 89 | 0.0192 | 1.23 |
| 1222 | ACTB_HUMAN | Actin beta | 7p22 | 42 | 5.20 | 30 | 13/99 | 115 | 0.0232 | 0.72 |
| 335 | ALBU_HUMAN | Albumin | 4q13.3 | 71 | 5.90 | 38 | 19/33 | 188 | 0.0003 | 0.56 |
| 337 | ALBU_HUMAN | Albumin | 4q13.3 | 71 | 5.90 | 41 | 23/63 | 156 | 0.0006 | 0.56 |
| 1129 | ALBU_HUMAN | Albumin | 4q13.3 | 71 | 5.90 | 31 | 16/55 | 104 | <0.0001 | 0.60 |
| 1144 | ALBU_HUMAN | Albumin | 4q13.3 | 71 | 5.90 | 53 | 25/65 | 194 | 0.0003 | 0.63 |
| 1145 | ALBU_HUMAN | Albumin | 4q13.3 | 71 | 5.90 | 46 | 22/54 | 185 | 0.0002 | 0.59 |
| 1155 | ALBU_HUMAN | Albumin | 4q13.3 | 71 | 5.90 | 48 | 28/65 | 217 | 0.0015 | 0.59 |
| 1210 | ALBU_HUMAN | Albumin | 4q13.3 | 71 | 5.90 | 29 | 18/41 | 133 | 0.0211 | 0.67 |
| 462 | ALDH2_HUMAN | Aldehyde dehydrogenase 2 family (mitochondrial) | 12q24.2 | 57 | 6.80 | 28 | 11/42 | 82 | 0.0345 | 1.25 |
| 1143 | ALDH2_HUMAN | Aldehyde dehydrogenase 2 family (mitochondrial) | 12q24.2 | 57 | 6.80 | 30 | 12/37 | 102 | 0.0001 | 1.60 |
| 1490 | ANXA2_HUMAN | Annexin A2 | 15q22.2 | 39 | 8.50 | 16 | 6/19 | 57 | 0.0561 | 1.28 |
| 1180 | ANXA3_HUMAN | Annexin A3 | 4q21.21 | 37 | 5.50 | 31 | 13/50 | 110 | 0.0129 | 1.28 |
| 1186 | ANXA5_HUMAN | Annexin A5 | 4q27 | 36 | 4.80 | 32 | 9/60 | 60 | 0.0023 | 0.63 |
| 919 | APT_HUMAN | Adenine phosphoribosyltransferase | 16q24 | 20 | 5.70 | 31 | 4/9 | 77 | 0.0122 | 0.34 |
| 495 | ARP3_HUMAN | Actin-related Protein 3, ACTR | 2q14.1 | 48 | 5.50 | 28 | 11/31 | 113 | 0.0041 | 1.18 |
| 118 | ATPB_HUMAN | ATP synthase subunit beta (mitochondrial) *1 | 12p13.3 | 57 | 5.10 | 26 | 9/18 | 88 | 0.0216 | 1.53 |
| 786 | CATD_HUMAN | Cathepsin D | 11p15.5 | 45 | 6.10 | 26 | 14/43 | 94 | 0.0157 | 0.71 |
| 393 | CH60_HUMAN | Chaperonin, HSPD1, GROEL, HSP60 | 2q33.1 | 61 | 5.60 | 20 | 9/32 | 59 | 0.0284 | 1.47 |
| 401 | CH60_HUMAN | Chaperonin, HSPD1, GROEL, HSP60 | 2q33.1 | 61 | 5.60 | 44 | 23/84 | 128 | 0.0016 | 1.77 |
| 405 | CH60_HUMAN | Chaperonin, HSPD1, GROEL, HSP60 | 2q33.1 | 61 | 5.60 | 31 | 18/95 | 60 | 0.0009 | 2.01 |
| 1140 | CH60_HUMAN | Chaperonin, HSPD1, GROEL, HSP60 | 2q33.1 | 61 | 5.60 | 38 | 14/49 | 97 | <0.0001 | 1.97 |
| 1141 | CH60_HUMAN | Chaperonin, HSPD1, GROEL, HSP60 | 2q33.1 | 61 | 5.60 | 9 | 3/3 | 90 | <0.0001 | 2.10 |
| 1169 | CH60_HUMAN | Chaperonin, HSPD1, GROEL, HSP60 | 2q33.1 | 61 | 5.60 | 9 | 3/18 | 63 | 0.0024 | 1.79 |
| 863 | COMT_HUMAN | Catechol-O-methyltransferase | 22q11.21 | 30 | 5.20 | 30 | 7/46 | 59 | 0.0076 | 1.62 |
| 671 | CYTSA_HUMAN | Specc1L | 22q11.23 | 125 | 5.40 | 15 | 15/45 | 58 | 0.0330 | 0.83 |
| 1196 | DYST_HUMAN | Dystonin | 6p12.1 | 865 | 5.00 | 9 | 54/85 | 92 | 0.0254 | 0.76 |
| 798 | ECHM_HUMAN | Mitochondrial enoyl-CoA hydratase,short chain 1 (mitochondrial) | 10q26.2-q26.3 | 32 | 9.40 | 26 | 6/27 | 59 | 0.0039 | 0.70 |
| 114 | GANAB_HUMAN | Glucosidase alpha neutral | 11q12.3 | 107 | 5.70 | 15 | 13/32 | 66 | 0.0234 | 1.23 |
| 1158 | GCC2_HUMAN | GRIP and coiled-coil domain containing 2 | 2q12.3 | 197 | 5.00 | 10 | 14/34 | 59 | 0.0070 | 0.64 |
| 1160 | GRP78_HUMAN | Heat shock 70kDA protein (glucose regulated protein 78 kDA) | 9q33.3 | 72 | 4.90 | 38 | 22/102 | 112 | 0.0113 | 0.72 |
| 1161 | GRP78_HUMAN | Heat shock 70kDA protein (glucose regulated protein 78 kDA) | 9q33.3 | 72 | 4.90 | 24 | 12/45 | 72 | 0.0092 | 0.65 |
| 1162 | GRP78_HUMAN | Heat shock 70kDA protein (glucose regulated protein 78 kDA) | 9q33.3 | 72 | 4.90 | 44 | 26/78 | 180 | 0.0030 | 0.65 |
| 478 | GSHB_HUMAN | Glutathione synthetase | 20q11.2 | 53 | 5.60 | 21 | 9/27 | 71 | 0.0005 | 1.55 |
| 790 | GSTO1_HUMAN | Glutathione S-transferase omega 1 | 10q25.1 | 28 | 6.30 | 29 | 8/31 | 72 | 0.0491 | 0.85 |
| 1175 | GSTP1_HUMAN | Glutathione S-transferase pi 1 | 11q13 | 24 | 5.30 | 55 | 10/39 | 115 | 0.0239 | 0.69 |
| 1176 | GSTP1_HUMAN | Glutathione S-transferase pi 1 | 11q13 | 24 | 5.30 | 40 | 6/24 | 70 | 0.0098 | 0.77 |
| 862 | GSTP1_HUMAN | Glutathione S-transferase pi 1 | 11q3 | 24 | 5.30 | 39 | 6/26 | 67 | 0.0319 | 0.76 |
| 645 | HNRPC_HUMAN | Heterogenous nuclear ribonucleoprotein C | 14q11.2 | 34 | 4.80 | 12 | 4/31 | 80 | 0.0124 | 1.37 |
| 205 | HS90B_HUMAN | Heat shock protein 90kDa alpha, class B Member | 6p12 | 84 | 4.80 | 17 | 13/20 | 205 | 0.0130 | 1.97 |
| 208 | HS90B_HUMAN | Heat shock protein 90kDa alpha, class B Member | 6p12 | 84 | 4.80 | 16 | 9/23 | 69 | 0.0156 | 1.76 |
| 212 | HS90B_HUMAN | Heat shock protein 90kDa alpha, class B Member | 6p12 | 84 | 4.80 | 26 | 15/47 | 86 | 0.0057 | 2.07 |
| 1242 | HS90B_HUMAN | Heat shock protein 90kDa alpha, class B Member | 6p12 | 84 | 4.80 | 34 | 30/95 | 139 | 0.0318 | 1.71 |
| 1152 | HSP7C_HUMAN | Heat shock cognate, 71kDa protein | 11q24.1 | 71 | 5.20 | 38 | 27/98 | 124 | 0.0014 | 1.27 |
| 319 | HSP7C_HUMAN | Heat shock cognate, 71kDa protein | 11q24.1 | 71 | 5.20 | 29 | 18/39 | 240 | 0.0181 | 1.18 |
| 1261 | HSPB1_HUMAN | Heat shock, 27 kDA | 7q11.23 | 23 | 6.00 | 17 | 3/5 | 90 | 0.0069 | 0.71 |
| 716 | IPYR_HUMAN | Inorganic pyrophosphatase | 10q11.1- q24 | 33 | 5.50 | 34 | 11/76 | 60 | 0.0233 | 1.35 |
| 1168 | K1C16_HUMAN | Keratin 16 | 17q21.2 | 52 | 4.80 | 38 | 16/72 | 111 | 0.0011 | 1.48 |
| 1154 | K1C19_HUMAN | Keratin 19 | 17q21.2 | 44 | 4.90 | 50 | 20/108 | 104 | 0.0070 | 0.75 |
| 758 | MARE1_HUMAN | Microtubule-associated protein EB1 | 20q11.1-q11.23 | 30 | 4.90 | 46 | 8/40 | 68 | 0.0005 | 1.65 |
| 98 | MVP_HUMAN | Major vault protein | 6p11.2 | 100 | 5.20 | 25 | 20/55 | 137 | 0.0136 | 1.18 |
| 101 | MVP_HUMAN | Major vault protein | 6p11.2 | 100 | 5.20 | 17 | 10/21 | 87 | 0.0095 | 1.31 |
| 973 | NDKA_HUMAN | nucleoside diphosphate kinase 1 | 17q21.3 | 17 | 5.80 | 57 | 9/46 | 80 | 0.0315 | 1.44 |
| 976 | NDKA_HUMAN | nucleoside diphosphate kinase 1 | 17q21.3 | 17 | 5.80 | 44 | 9/28 | 72 | 0.0200 | 1.45 |
| 831 | NDUS3_HUMAN | NADH dehydrogenase | 11p11.11 | 30 | 7.80 | 43 | 12/97 | 84 | 0.0424 | 0.81 |
| 502 | OPA1_HUMAN | Optic atrophy 1 | 3q28-q29 | 112 | 8.60 | 9 | 8/13 | 57 | 0.0259 | 1.22 |
| 891 | PRDX3_HUMAN | Peroxiredoxin 3 | 10q25-q26 | 28 | 8.90 | 35 | 9/44 | 77 | 0.0493 | 1.25 |
| 1478 | RSSA_HUMAN | Ribosomal protein SA | 3p22.2 | 33 | 4.60 | 18 | 5/19 | 52 | 0.0385 | 1.36 |
| 476 | RUVB2_HUMAN | RuvB-like 2 protein, putative | 19p13.3 | 51 | 5.40 | 50 | 19/62 | 135 | 0.0181 | 1.31 |
| 542 | SAHH_HUMAN | S-adenosyl-L-homocysteine hydrolase | 20q11.22 | 48 | 5.90 | 21 | 10/43 | 66 | 0.0013 | 1.42 |
| 559 | SUCB1_HUMAN | Dihydrolipamide succinyltransferase | 13q12.2-q13.3 | 51 | 7.70 | 19 | 8/32 | 56 | 0.0460 | 1.27 |
| 1190 | TBA1B_HUMAN | Tubulin, alpha 1b | 12q13.12 | 51 | 4.80 | 52 | 23/108 | 105 | 0.0073 | 1.27 |
| 1202 | TBA1B_HUMAN | Tubulin, alpha 1b | 12q13.12 | 51 | 4.80 | 60 | 25/86 | 159 | 0.0111 | 1.41 |
| 1146 | TBB4B_HUMAN | Tubulin, beta 4B | 9q34 | 51 | 4.60 | 45 | 25/99 | 130 | 0.0011 | 1.27 |
| 1147 | TBB4B_HUMAN | Tubulin, beta 4B | 9q34 | 50 | 4.60 | 68 | 42/107 | 241 | 0.0014 | 1.43 |
| 1189 | U520_HUMAN | Small nuclear ribonucleportotein | 2q11.2 | 246 | 5.70 | 12 | 16/41 | 58 | 0.0051 | 1.53 |
| 104 | UBA1_HUMAN | Ubiquitin-like modifier activation Enzyme 1 | Xp11.23 | 119 | 5.40 | 18 | 16/60 | 77 | 0.0220 | 0.83 |

MW, Molecular weight; pI, isoelectric point

* Chromosomal location was added according to information provided by NCBI database (http://www.ncbi.nlm.nih.gov/gene; February 2016).

** Fold-change shows the regulation of the protein: Values >1 represent a higher level of proteins in SCC while values <1 show a lower level in SCC samples compared to UCC samples.
